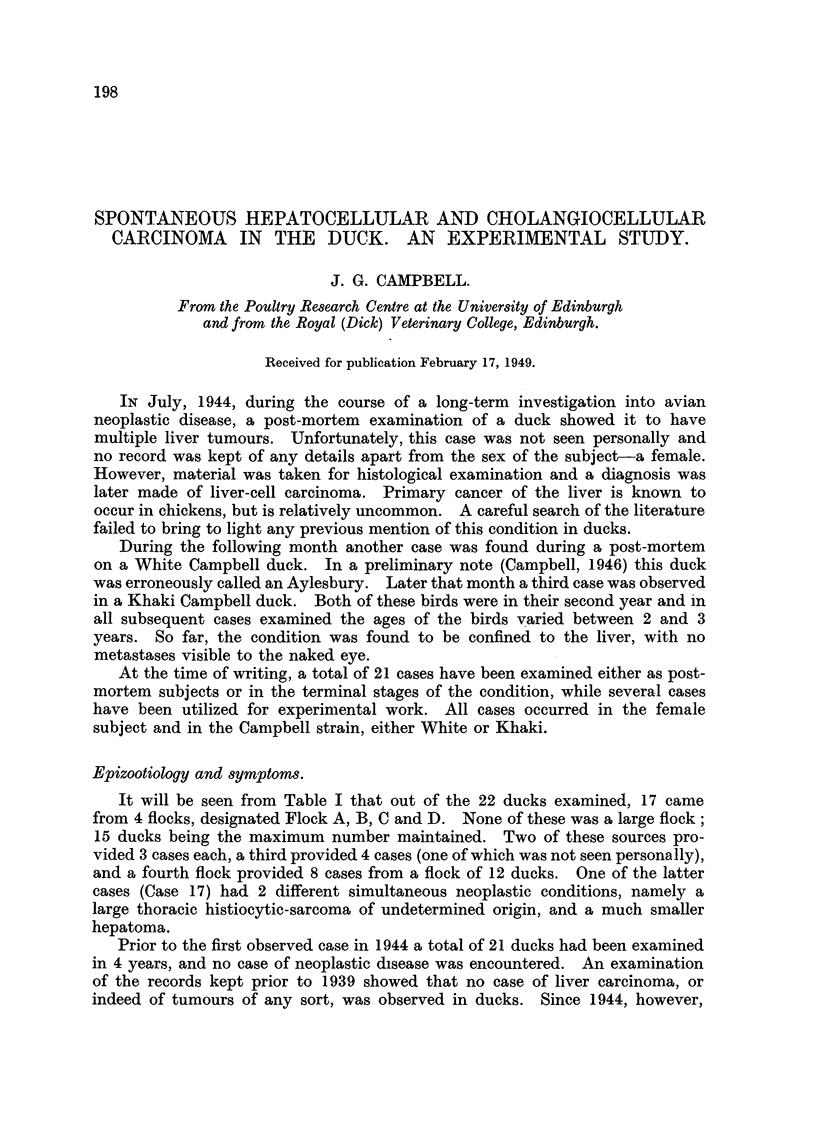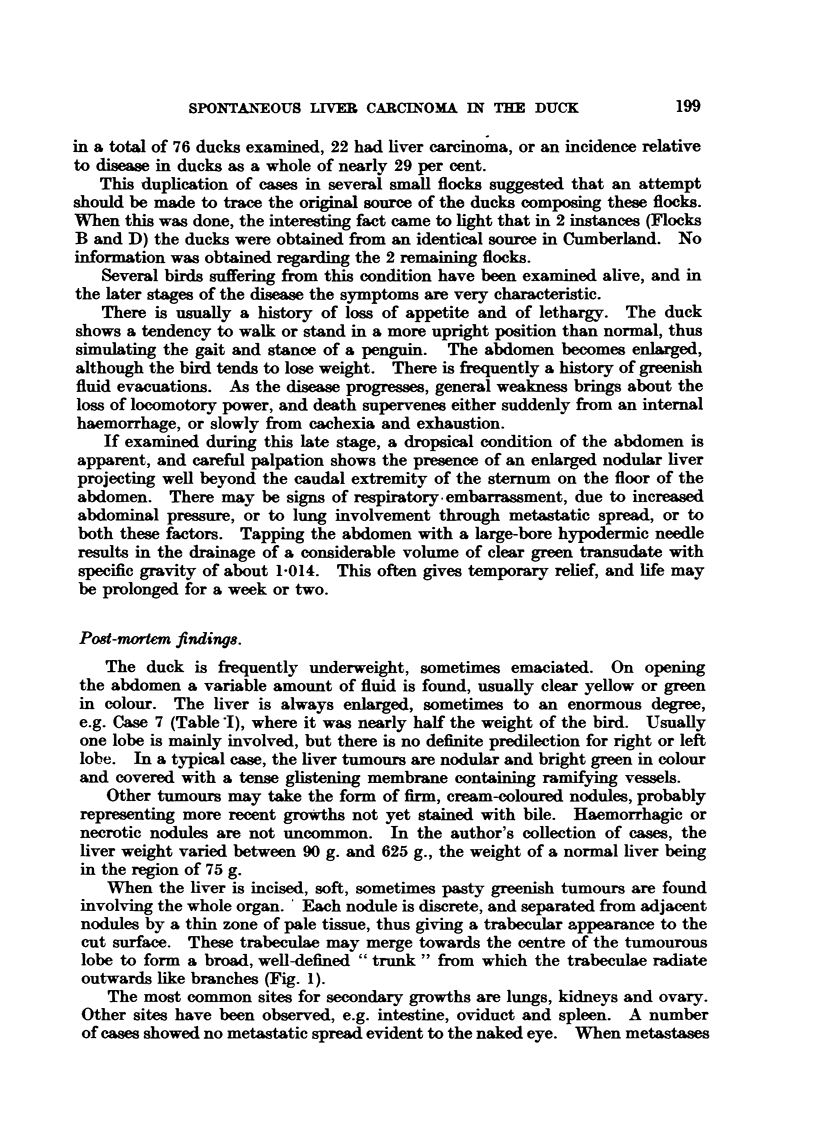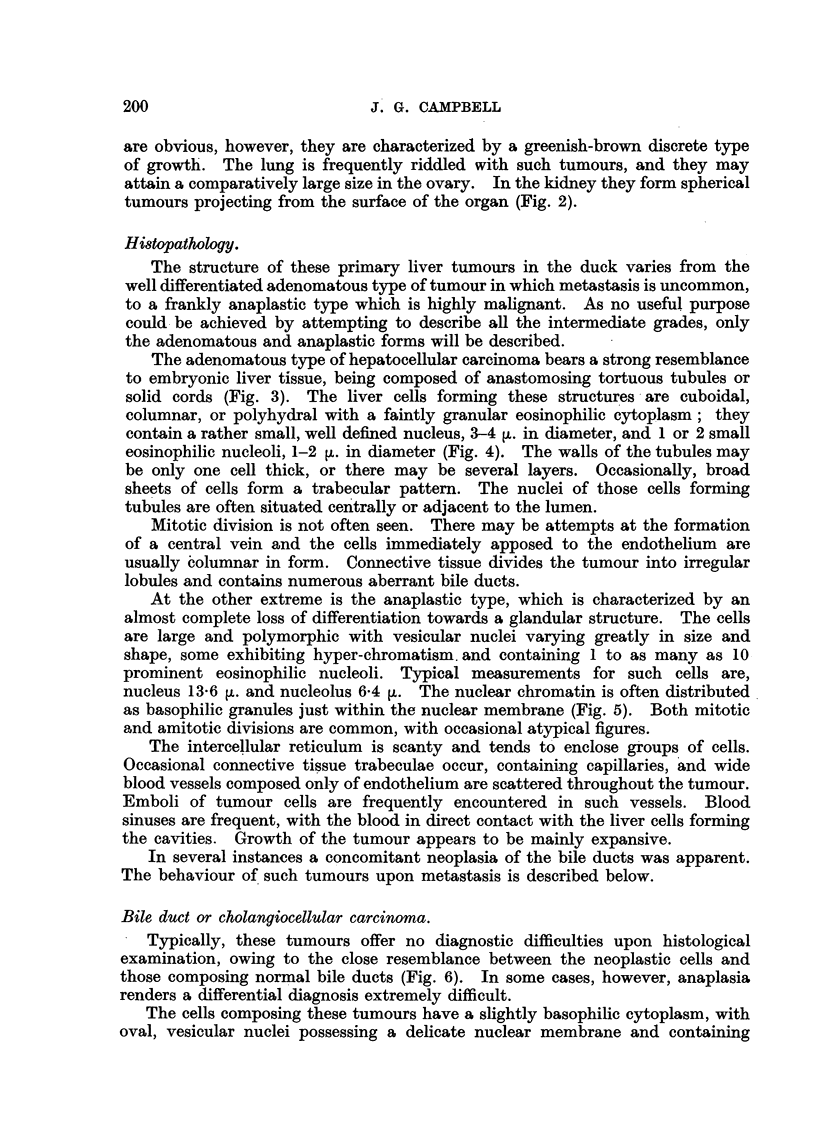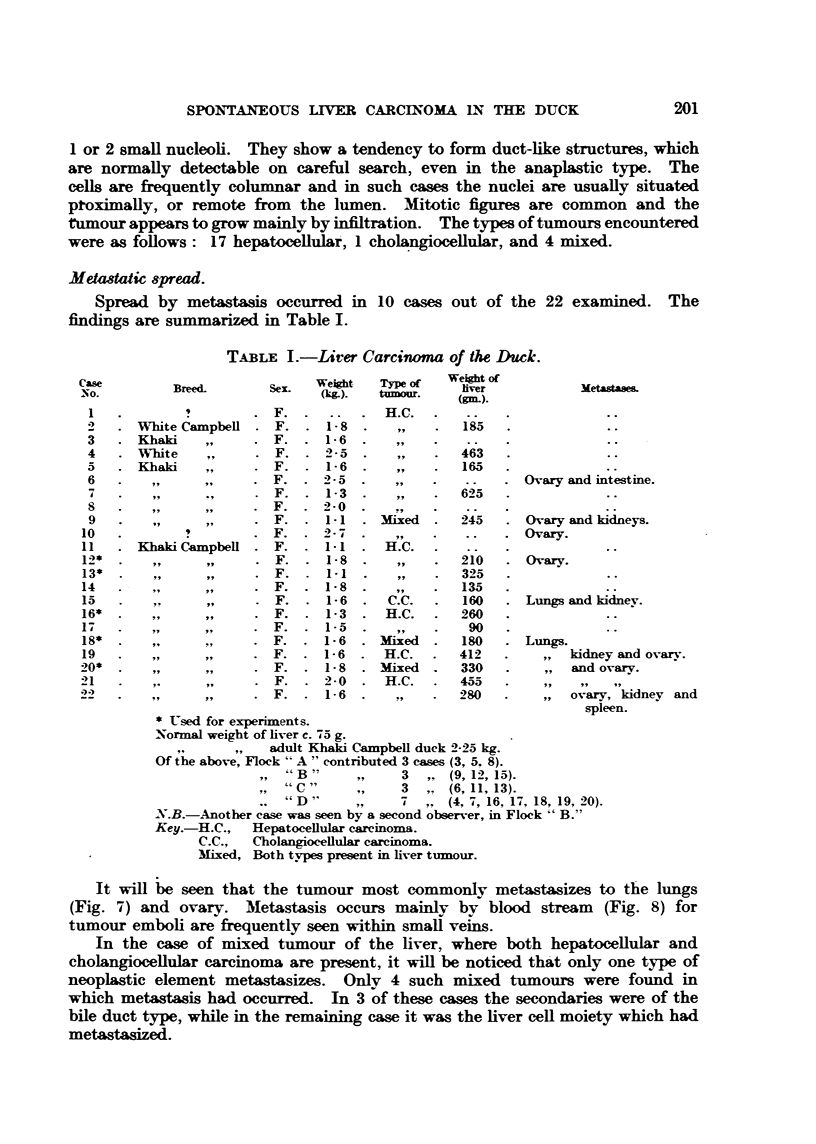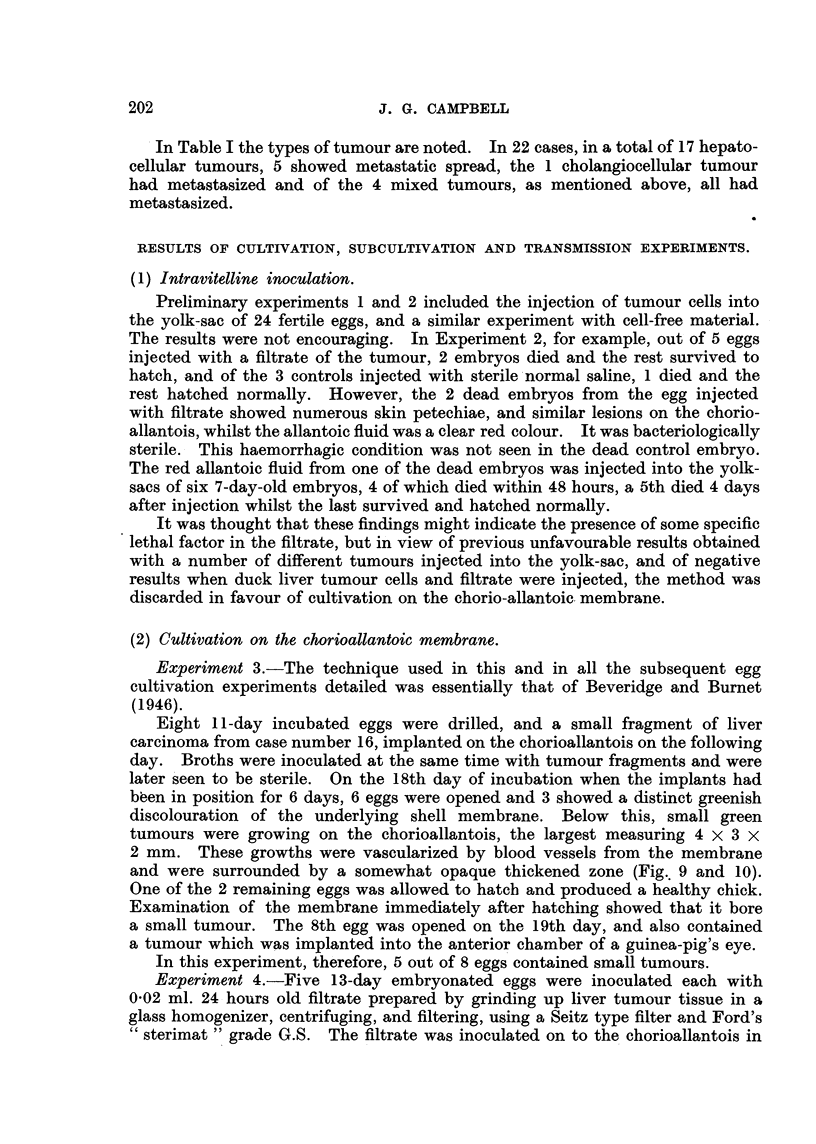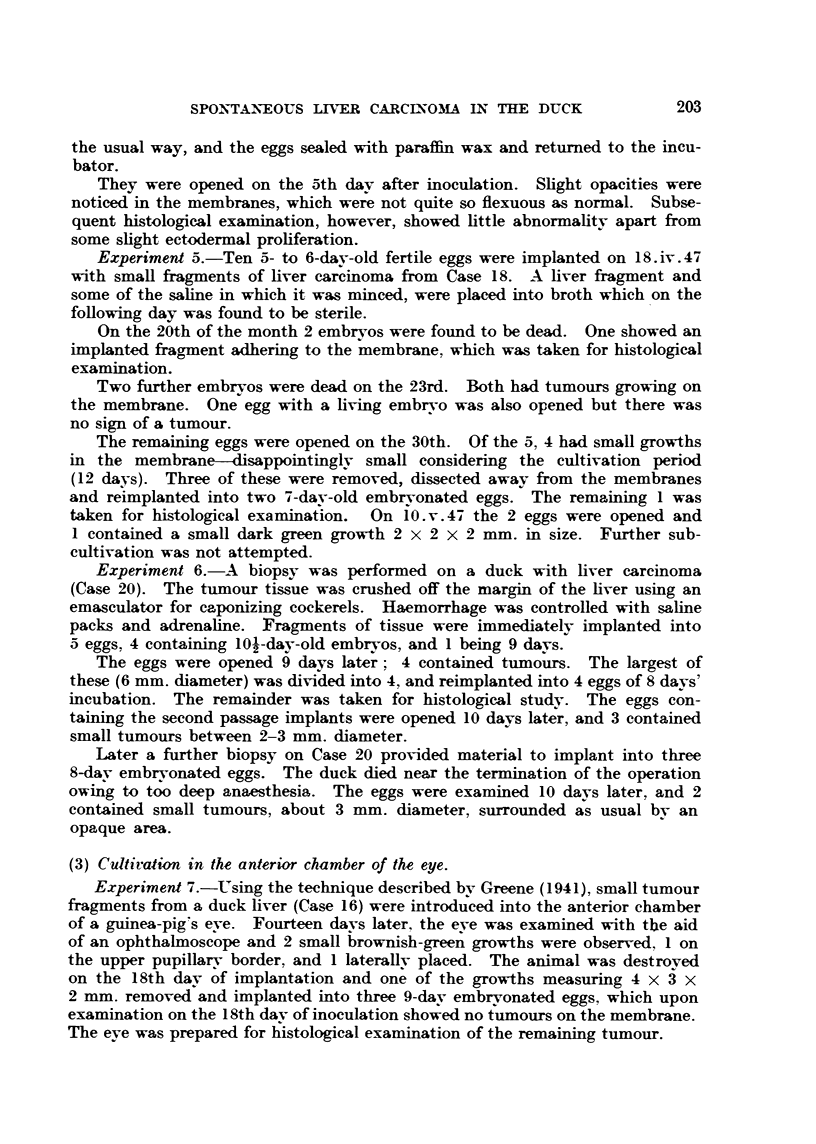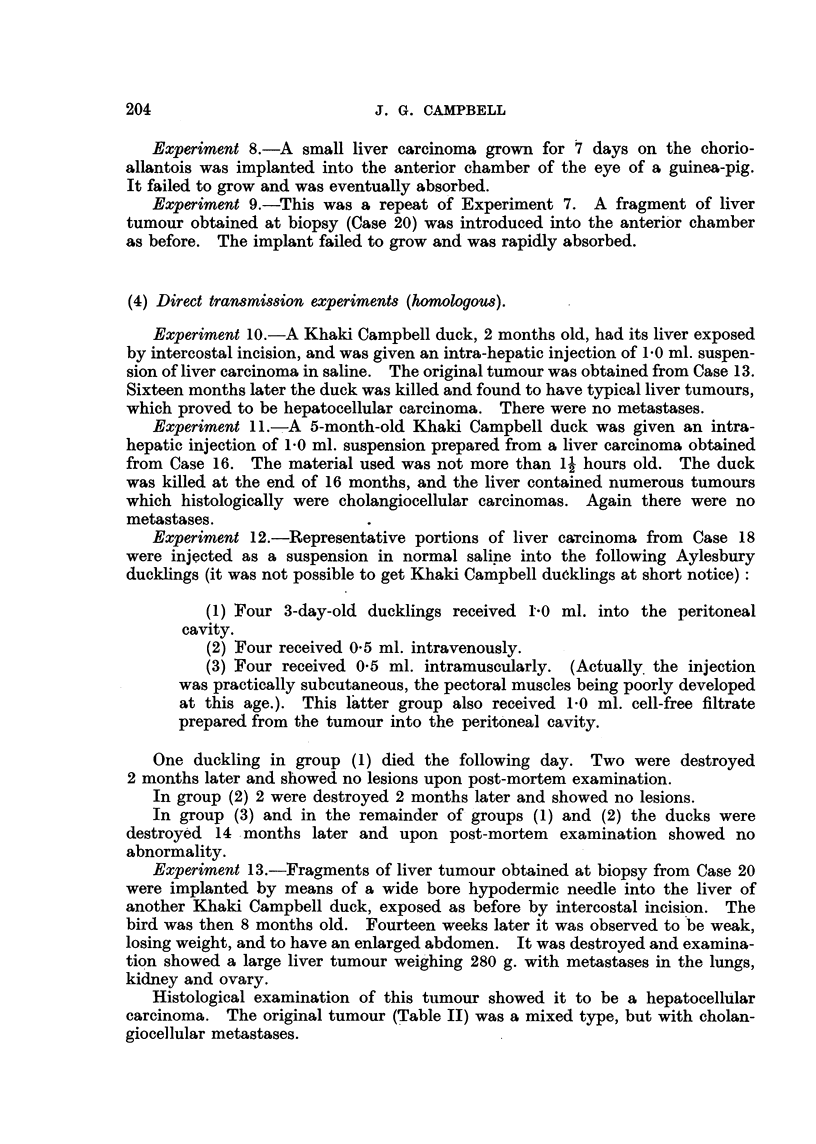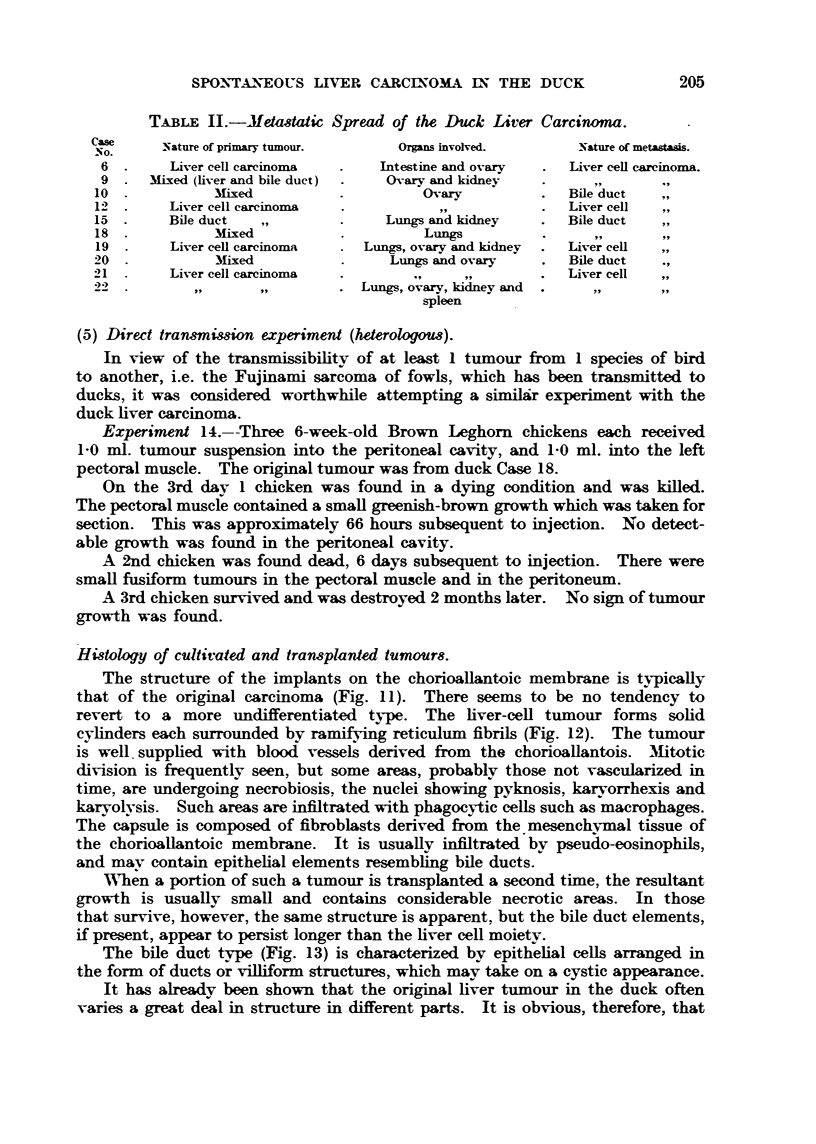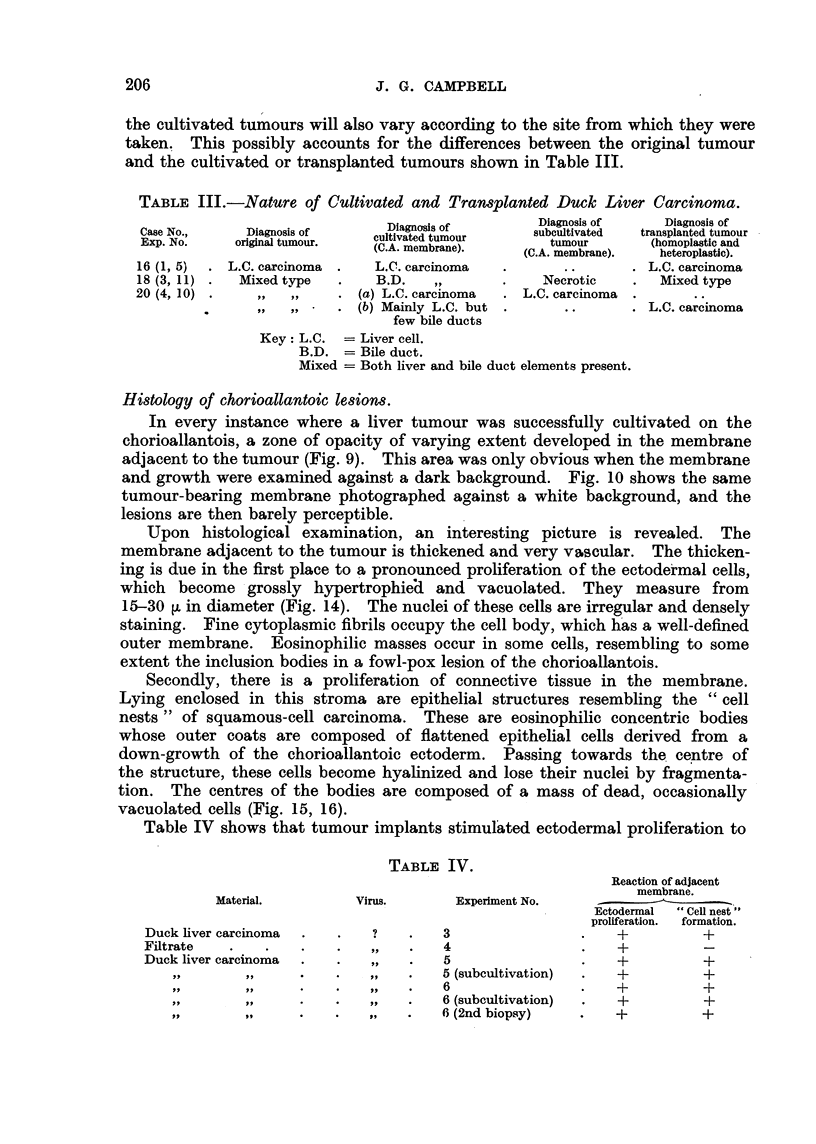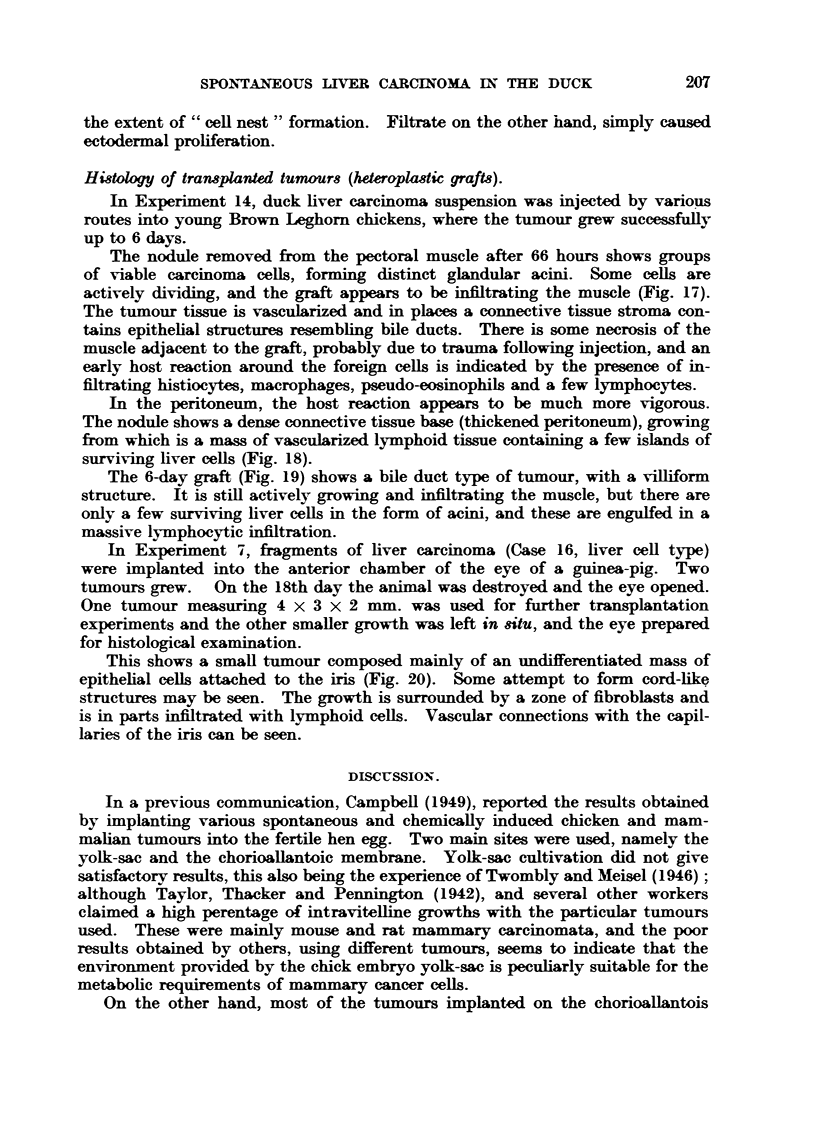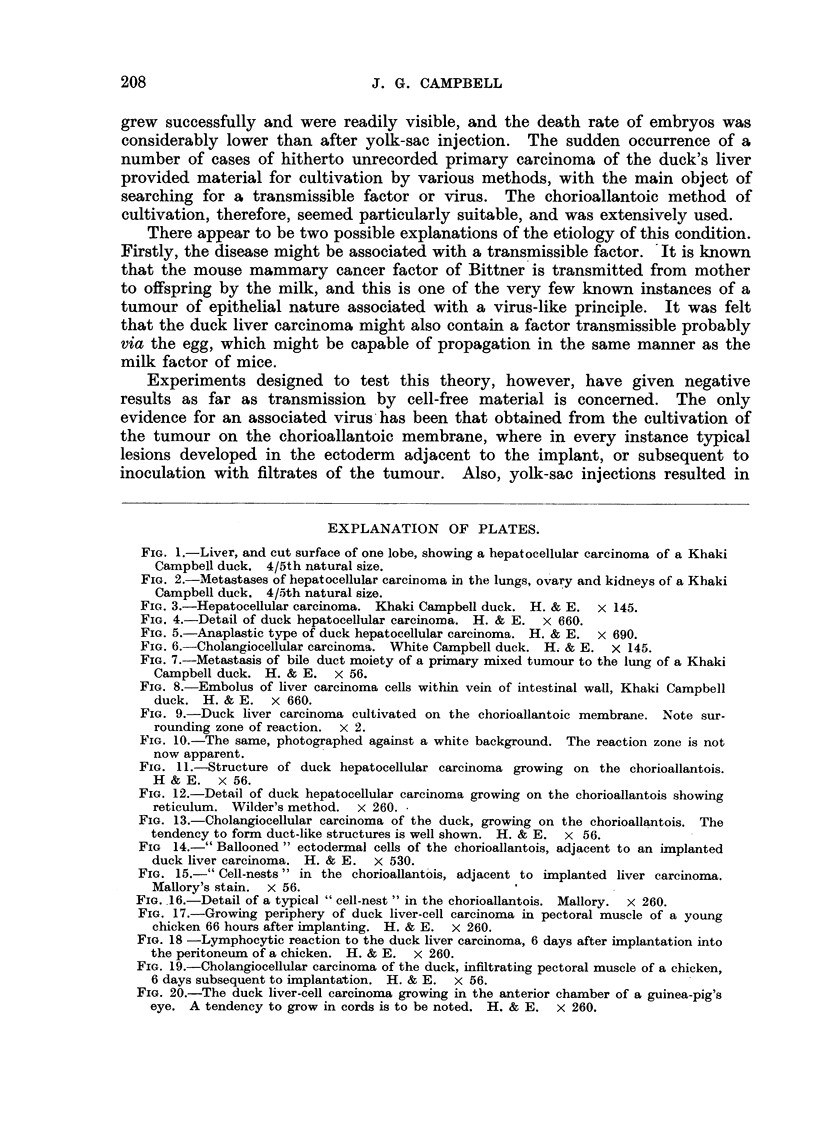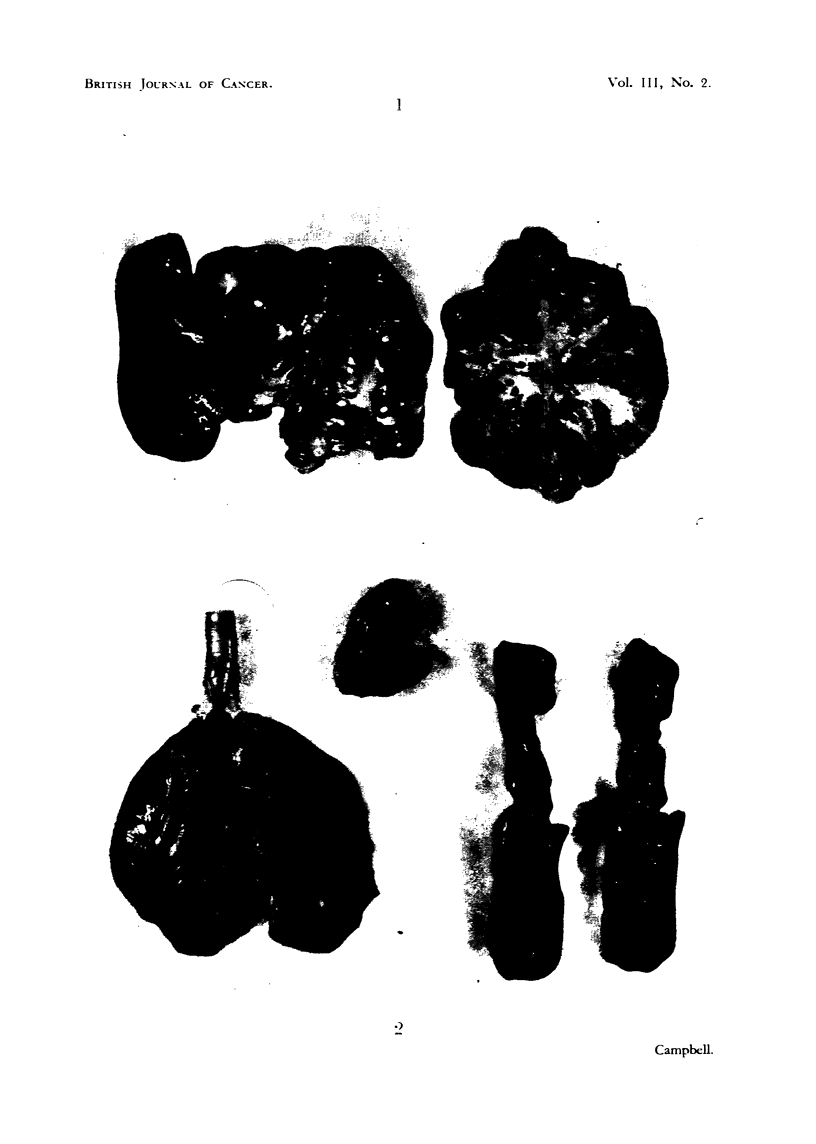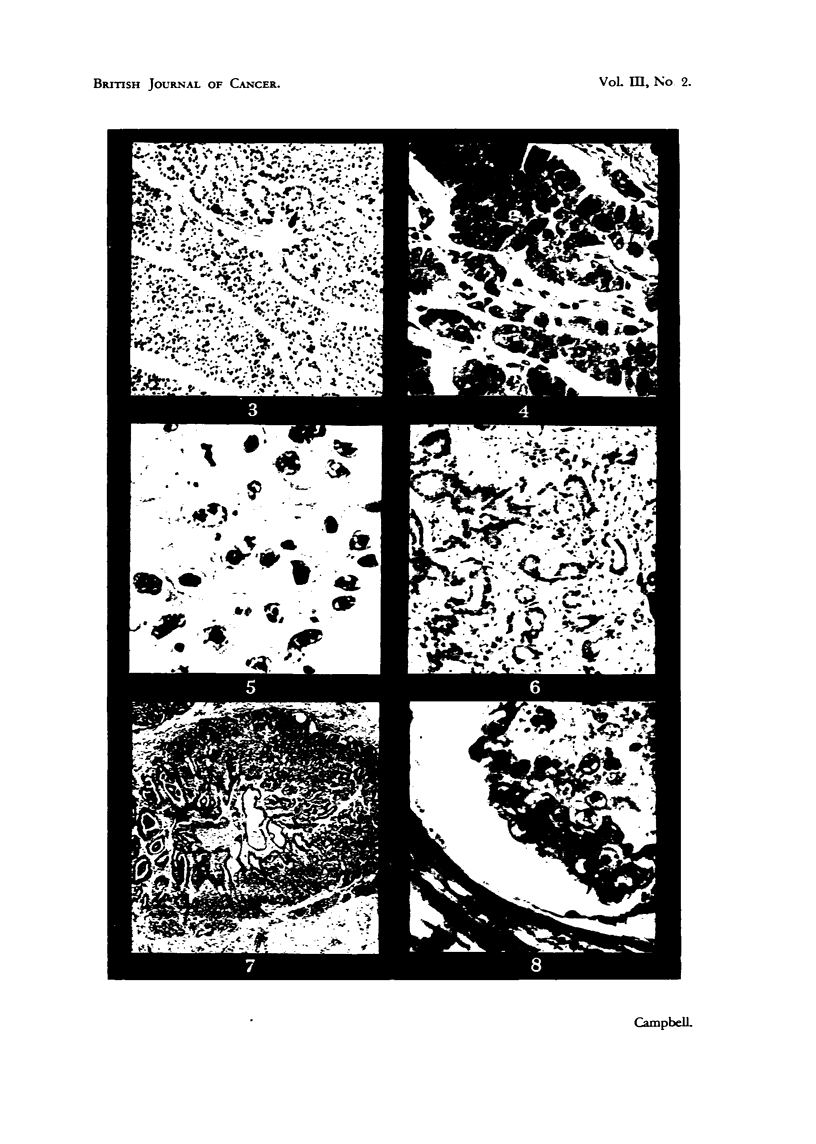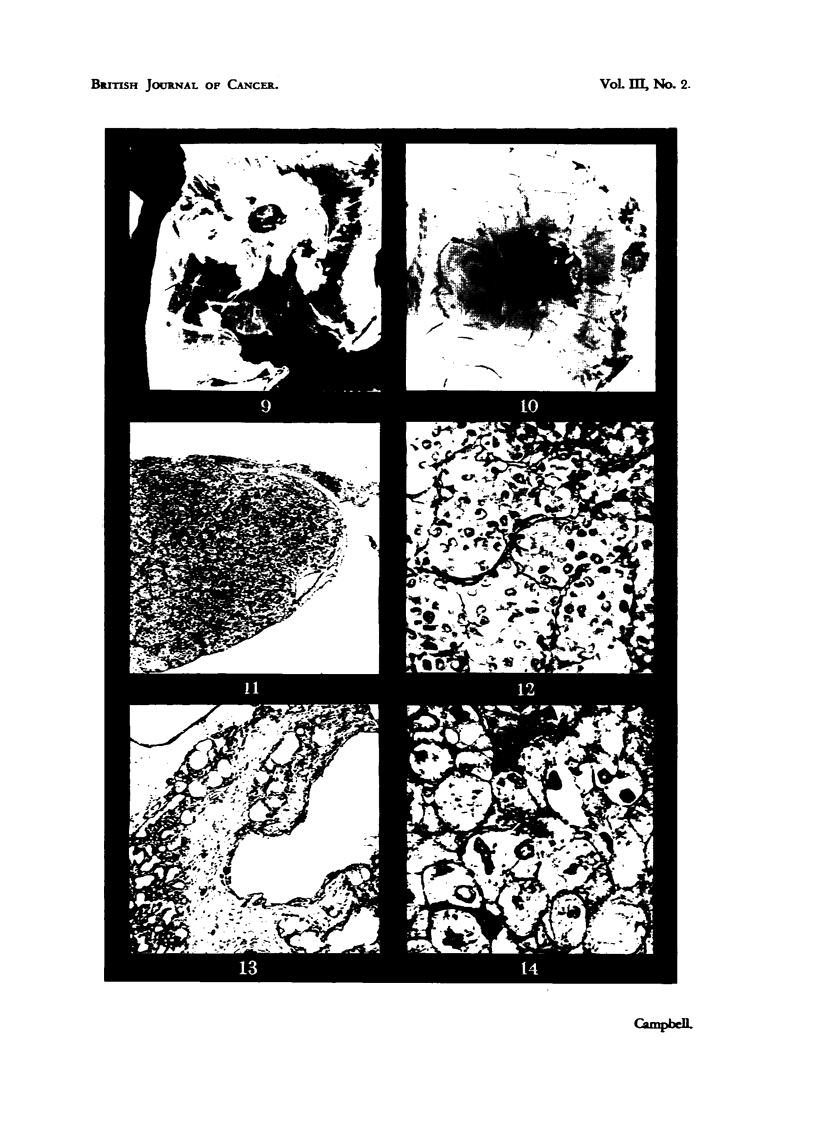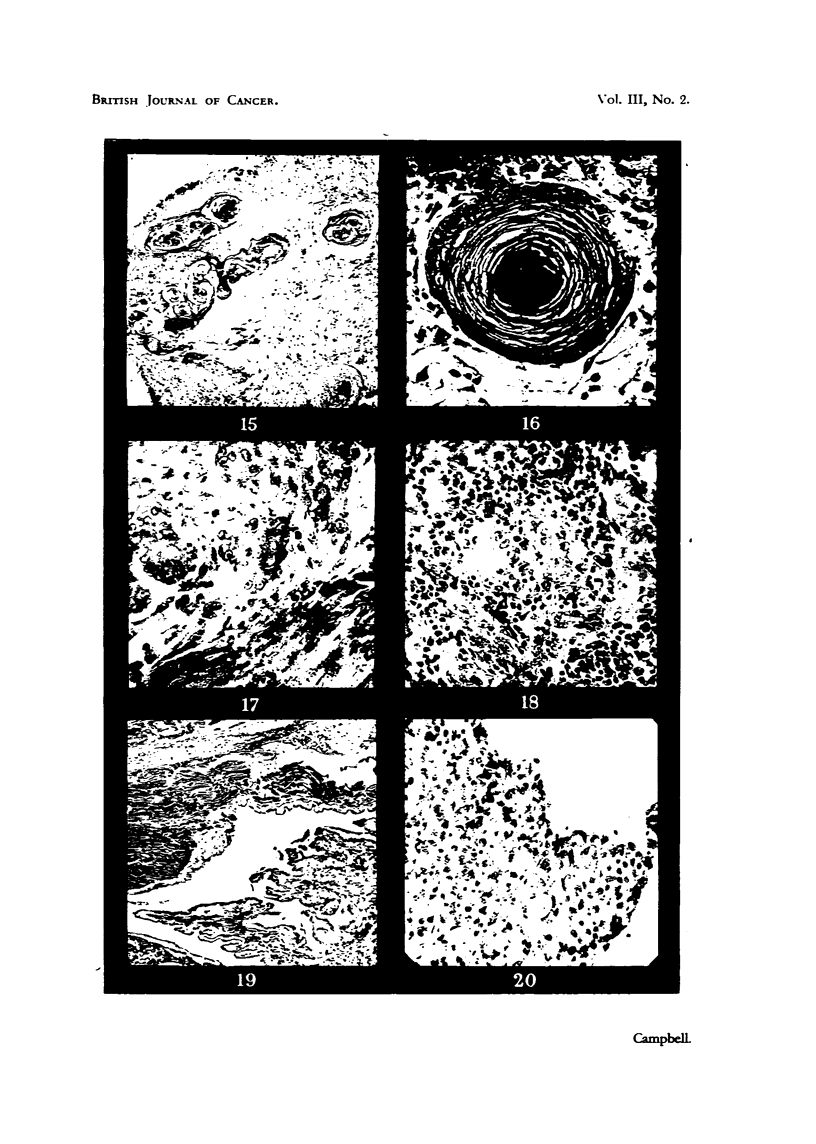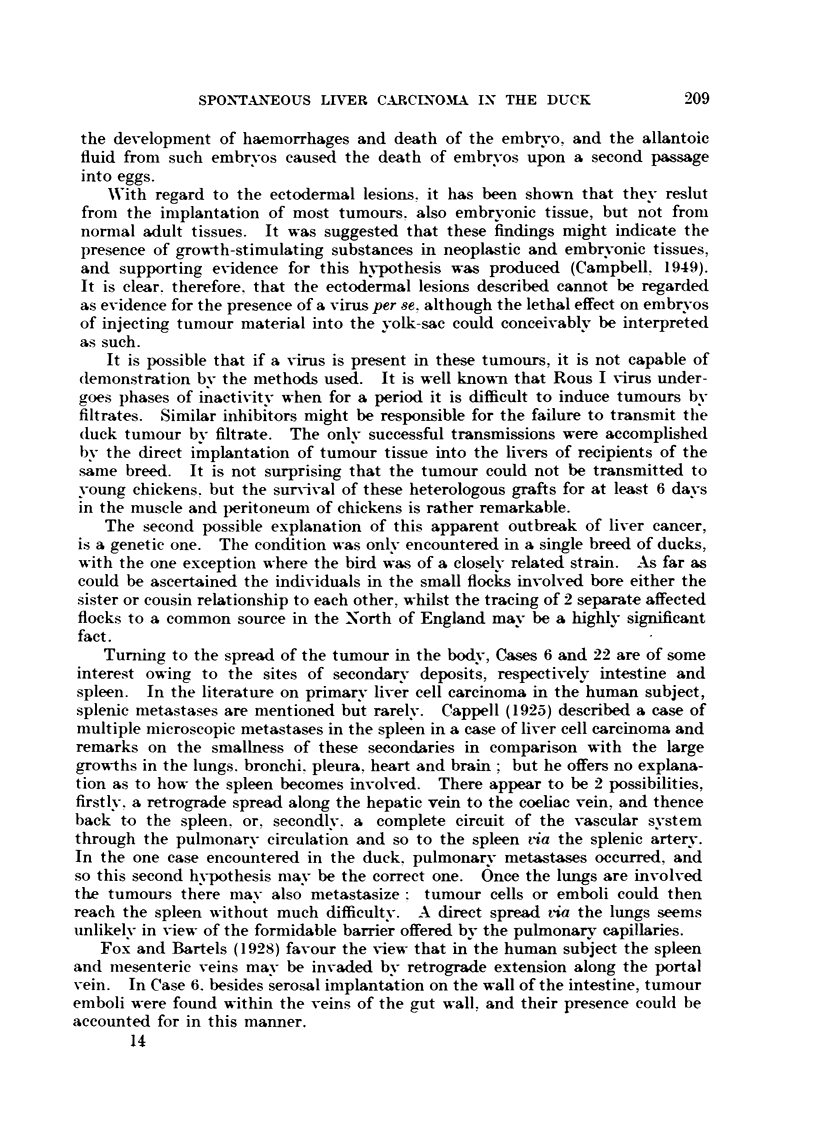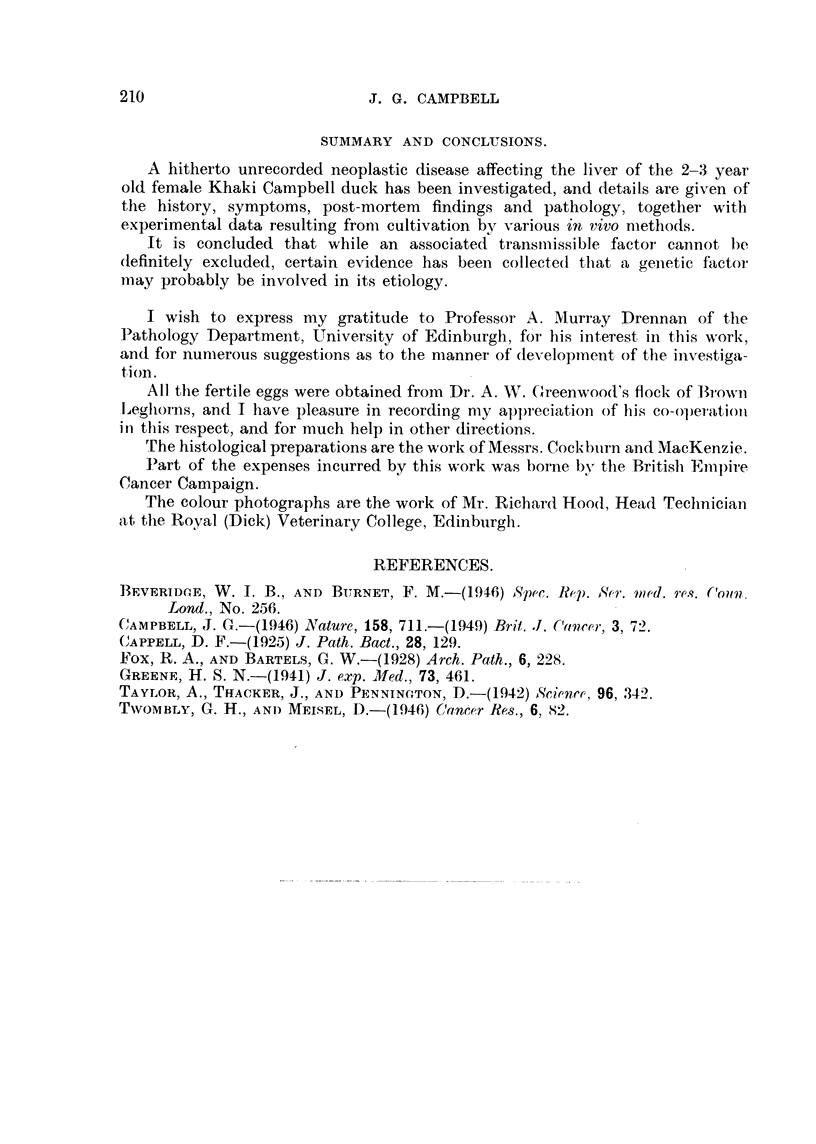# Spontaneous Hepatocellular and Cholangiocellular Carcinoma in the Duck. An Experimental Study

**DOI:** 10.1038/bjc.1949.21

**Published:** 1949-06

**Authors:** J. G. Campbell

## Abstract

**Images:**


### SPONTANEOUS HEPATOCELLULAR AND CHOLANGIOCELLULAR CARCINOMA IN THE DUCK. AN EXPERIMENTAL STUDY.

J. G. CAMPBELL.

*From the Poultry Research Centre at the University of Edinburgh and from the Royal (Dick) Veterinary College, Edinburgh.*

Received for publication February 17, 1949.

In July, 1944, during the course of a long-term investigation into avian neoplastic disease, a post-mortem examination of a duck showed it to have multiple liver tumours. Unfortunately, this case was not seen personally and no record was kept of any details apart from the sex of the subject—a female. However, material was taken for histological examination and a diagnosis was later made of liver-cell carcinoma. Primary cancer of the liver is known to occur in chickens, but is relatively uncommon. A careful search of the literature failed to bring to light any previous mention of this condition in ducks.

During the following month another case was found during a post-mortem on a White Campbell duck. In a preliminary note (Campbell, 1946) this duck was erroneously called an Aylesbury. Later that month a third case was observed in a Khaki Campbell duck. Both of these birds were in their second year and in all subsequent cases examined the ages of the birds varied between 2 and 3 years. So far, the condition was found to be confined to the liver, with no metastases visible to the naked eye.

At the time of writing, a total of 21 cases have been examined either as post-mortem subjects or in the terminal stages of the condition, while several cases have been utilized for experimental work. All cases occurred in the female subject and in the Campbell strain, either White or Khaki.

*Epizootiology and symptoms.*

It will be seen from Table I that out of the 22 ducks examined, 17 came from 4 flocks, designated Flock A, B, C and D. None of these was a large flock ; 15 ducks being the maximum number maintained. Two of these sources provided 3 cases each, a third provided 4 cases (one of which was not seen personally), and a fourth flock provided 8 cases from a flock of 12 ducks. One of the latter cases (Case 17) had 2 different simultaneous neoplastic conditions, namely a large thoracic histiocytic-sarcoma of undetermined origin, and a much smaller hepatoma.

Prior to the first observed case in 1944 a total of 21 ducks had been examined in 4 years, and no case of neoplastic disease was encountered. An examination of the records kept prior to 1939 showed that no case of liver carcinoma, or indeed of tumours of any sort, was observed in ducks. Since 1944, however, in a total of 76 ducks examined, 22 had liver carcinoma, or an incidence relative to disease in ducks as a whole of nearly 29 per cent.

This duplication of cases in several small flocks suggested that an attempt should be made to trace the original source of the ducks composing these flocks. When this was done, the interesting fact came to light that in 2 instances (Flocks B and D) the ducks were obtained from an identical source in Cumberland. No information was obtained regarding the 2 remaining flocks.

Several birds suffering from this condition have been examined alive, and in the later stages of the disease the symptoms are very characteristic.

There is usually a history of loss of appetite and of lethargy. The duck shows a tendency to walk or stand in a more upright position than normal, thus simulating the gait and stance of a penguin. The abdomen becomes enlarged, although the bird tends to lose weight. There is frequently a history of greenish fluid evacuations. As the disease progresses, general weakness brings about the loss of locomotory power, and death supervenes either suddenly from an internal haemorrhage, or slowly from cachexia and exhaustion.

If examined during this late stage, a dropsical condition of the abdomen is apparent, and careful palpation shows the presence of an enlarged nodular liver projecting well beyond the caudal extremity of the sternum on the floor of the abdomen. There may be signs of respiratory embarrassment, due to increased abdominal pressure, or to lung involvement through metastatic spread, or to both these factors. Tapping the abdomen with a large-bore hypodermic needle results in the drainage of a considerable volume of clear green transudate with specific gravity of about 1·014. This often gives temporary relief, and life may be prolonged for a week or two.

*Post-mortem findings.*

The duck is frequently underweight, sometimes emaciated. On opening the abdomen a variable amount of fluid is found, usually clear yellow or green in colour. The liver is always enlarged, sometimes to an enormous degree, e.g. Case 7 (Table I), where it was nearly half the weight of the bird. Usually one lobe is mainly involved, but there is no definite predilection for right or left lobe. In a typical case, the liver tumours are nodular and bright green in colour and covered with a tense glistening membrane containing ramifying vessels.

Other tumours may take the form of firm, cream-coloured nodules, probably representing more recent growths not yet stained with bile. Haemorrhagic or necrotic nodules are not uncommon. In the author's collection of cases, the liver weight varied between 90 g. and 625 g., the weight of a normal liver being in the region of 75 g.

When the liver is incised, soft, sometimes pasty greenish tumours are found involving the whole organ. Each nodule is discrete, and separated from adjacent nodules by a thin zone of pale tissue, thus giving a trabecular appearance to the cut surface. These trabeculae may merge towards the centre of the tumourous lobe to form a broad, well-defined " trunk " from which the trabeculae radiate outwards like branches (Fig. 1).

The most common sites for secondary growths are lungs, kidneys and ovary. Other sites have been observed, e.g. intestine, oviduct and spleen. A number of cases showed no metastatic spread evident to the naked eye. When metastases are obvious, however, they are characterized by a greenish-brown discrete type of growth. The lung is frequently riddled with such tumours, and they may attain a comparatively large size in the ovary. In the kidney they form spherical tumours projecting from the surface of the organ (Fig. 2).

*Histopathology.*

The structure of these primary liver tumours in the duck varies from the well differentiated adenomatous type of tumour in which metastasis is uncommon, to a frankly anaplastic type which is highly malignant. As no useful purpose could be achieved by attempting to describe all the intermediate grades, only the adenomatous and anaplastic forms will be described.

The adenomatous type of hepatocellular carcinoma bears a strong resemblance to embryonic liver tissue, being composed of anastomosing tortuous tubules or solid cords (Fig. 3). The liver cells forming these structures are cuboidal, columnar, or polyhydral with a faintly granular eosinophilic cytoplasm ; they contain a rather small, well defined nucleus, 3–4 μ. in diameter, and 1 or 2 small eosinophilic nucleoli, 1–2 μ. in diameter (Fig. 4). The walls of the tubules may be only one cell thick, or there may be several layers. Occasionally, broad sheets of cells form a trabecular pattern. The nuclei of those cells forming tubules are often situated centrally or adjacent to the lumen.

Mitotic division is not often seen. There may be attempts at the formation of a central vein and the cells immediately apposed to the endothelium are usually columnar in form. Connective tissue divides the tumour into irregular lobules and contains numerous aberrant bile ducts.

At the other extreme is the anaplastic type, which is characterized by an almost complete loss of differentiation towards a glandular structure. The cells are large and polymorphic with vesicular nuclei varying greatly in size and shape, some exhibiting hyper-chromatism and containing 1 to as many as 10 prominent eosinophilic nucleoli. Typical measurements for such cells are, nucleus 13·6 μ. and nucleolus 6·4 μ. The nuclear chromatin is often distributed as basophilic granules just within the nuclear membrane (Fig. 5). Both mitotic and amitotic divisions are common, with occasional atypical figures.

The intercellular reticulum is scanty and tends to enclose groups of cells. Occasional connective tissue trabeculae occur, containing capillaries, and wide blood vessels composed only of endothelium are scattered throughout the tumour. Emboli of tumour cells are frequently encountered in such vessels. Blood sinuses are frequent, with the blood in direct contact with the liver cells forming the cavities. Growth of the tumour appears to be mainly expansive.

In several instances a concomitant neoplasia of the bile ducts was apparent. The behaviour of such tumours upon metastasis is described below.

*Bile duct or cholangiocellular carcinoma.*

Typically, these tumours offer no diagnostic difficulties upon histological examination, owing to the close resemblance between the neoplastic cells and those composing normal bile ducts (Fig. 6). In some cases, however, anaplasia renders a differential diagnosis extremely difficult.

The cells composing these tumours have a slightly basophilic cytoplasm, with oval, vesicular nuclei possessing a delicate nuclear membrane and containing

1 or 2 small nucleoli. They show a tendency to form duct-like structures, which are normally detectable on careful search, even in the anaplastic type. The cells are frequently columnar and in such cases the nuclei are usually situated proximally, or remote from the lumen. Mitotic figures are common and the tumour appears to grow mainly by infiltration. The types of tumours encountered were as follows : 17 hepatocellular, 1 cholangiocellular, and 4 mixed.

*Metastatic spread.*

Spread by metastasis occurred in 10 cases out of the 22 examined. The findings are summarized in Table I.

TABLE I.—*Liver Carcinoma of the Duck.*

| Case No. | Breed. | Sex. | Weight (kg.). | Type of tumour. | Weight of liver (gm.). | Metastases. |
|---|---|---|---|---|---|---|
| 1 | ? | F. | .. | H.C. | .. | .. |
| 2 | White Campbell | F. | 1·8 | ,, | 185 | .. |
| 3 | Khaki ,, | F. | 1·6 | ,, | .. | .. |
| 4 | White ,, | F. | 2·5 | ,, | 463 | .. |
| 5 | Khaki ,, | F. | 1·6 | ,, | 165 | .. |
| 6 | ,, ,, | F. | 2·5 | ,, | .. | Ovary and intestine. |
| 7 | ,, ,, | F. | 1·3 | ,, | 625 | .. |
| 8 | ,, ,, | F. | 2·0 | ,, | .. | .. |
| 9 | ,, ,, | F. | 1·1 | Mixed | 245 | Ovary and kidneys. |
| 10 | ? | F. | 2·7 | ,, | .. | Ovary. |
| 11 | Khaki Campbell | F. | 1·1 | H.C. | .. | .. |
| 12* | ,, ,, | F. | 1·8 | ,, | 210 | Ovary. |
| 13* | ,, ,, | F. | 1·1 | ,, | 325 | .. |
| 14 | ,, ,, | F. | 1·8 | ,, | 135 | .. |
| 15 | ,, ,, | F. | 1·6 | C.C. | 160 | Lungs and kidney. |
| 16* | ,, ,, | F. | 1·3 | H.C. | 260 | .. |
| 17 | ,, ,, | F. | 1·5 | ,, | 90 | .. |
| 18* | ,, ,, | F. | 1·6 | Mixed | 180 | Lungs. |
| 19 | ,, ,, | F. | 1·6 | H.C. | 412 | ,, kidney and ovary. |
| 20* | ,, ,, | F. | 1·8 | Mixed | 330 | ,, and ovary. |
| 21 | ,, ,, | F. | 2·0 | H.C. | 455 | ,, ,, ,, |
| 22 | ,, ,, | F. | 1·6 | ,, | 280 | ,, ovary, kidney and spleen. |

* Used for experiments.
Normal weight of liver *c.* 75 g.
,, ,, adult Khaki Campbell duck 2·25 kg.
Of the above, Flock " A " contributed 3 cases (3, 5, 8).
,, " B " ,, 3 ,, (9, 12, 15).
,, " C " ,, 3 ,, (6, 11, 13).
,, " D " ,, 7 ,, (4, 7, 16, 17, 18, 19, 20).
*N.B.*—Another case was seen by a second observer, in Flock " B."
*Key.*—H.C., Hepatocellular carcinoma.
C.C., Cholangiocellular carcinoma.
Mixed, Both types present in liver tumour.

It will be seen that the tumour most commonly metastasizes to the lungs (Fig. 7) and ovary. Metastasis occurs mainly by blood stream (Fig. 8) for tumour emboli are frequently seen within small veins.

In the case of mixed tumour of the liver, where both hepatocellular and cholangiocellular carcinoma are present, it will be noticed that only one type of neoplastic element metastasizes. Only 4 such mixed tumours were found in which metastasis had occurred. In 3 of these cases the secondaries were of the bile duct type, while in the remaining case it was the liver cell moiety which had metastasized.

In Table I the types of tumour are noted. In 22 cases, in a total of 17 hepatocellular tumours, 5 showed metastatic spread, the 1 cholangiocellular tumour had metastasized and of the 4 mixed tumours, as mentioned above, all had metastasized.

### RESULTS OF CULTIVATION, SUBCULTIVATION AND TRANSMISSION EXPERIMENTS.

(1) *Intravitelline inoculation.*

Preliminary experiments 1 and 2 included the injection of tumour cells into the yolk-sac of 24 fertile eggs, and a similar experiment with cell-free material. The results were not encouraging. In Experiment 2, for example, out of 5 eggs injected with a filtrate of the tumour, 2 embryos died and the rest survived to hatch, and of the 3 controls injected with sterile normal saline, 1 died and the rest hatched normally. However, the 2 dead embryos from the egg injected with filtrate showed numerous skin petechiae, and similar lesions on the chorio-allantois, whilst the allantoic fluid was a clear red colour. It was bacteriologically sterile. This haemorrhagic condition was not seen in the dead control embryo. The red allantoic fluid from one of the dead embryos was injected into the yolk-sacs of six 7-day-old embryos, 4 of which died within 48 hours, a 5th died 4 days after injection whilst the last survived and hatched normally.

It was thought that these findings might indicate the presence of some specific lethal factor in the filtrate, but in view of previous unfavourable results obtained with a number of different tumours injected into the yolk-sac, and of negative results when duck liver tumour cells and filtrate were injected, the method was discarded in favour of cultivation on the chorio-allantoic membrane.

(2) *Cultivation on the chorioallantoic membrane.*

*Experiment* 3.—The technique used in this and in all the subsequent egg cultivation experiments detailed was essentially that of Beveridge and Burnet (1946).

Eight 11-day incubated eggs were drilled, and a small fragment of liver carcinoma from case number 16, implanted on the chorioallantois on the following day. Broths were inoculated at the same time with tumour fragments and were later seen to be sterile. On the 18th day of incubation when the implants had been in position for 6 days, 6 eggs were opened and 3 showed a distinct greenish discolouration of the underlying shell membrane. Below this, small green tumours were growing on the chorioallantois, the largest measuring 4 × 3 × 2 mm. These growths were vascularized by blood vessels from the membrane and were surrounded by a somewhat opaque thickened zone (Fig. 9 and 10). One of the 2 remaining eggs was allowed to hatch and produced a healthy chick. Examination of the membrane immediately after hatching showed that it bore a small tumour. The 8th egg was opened on the 19th day, and also contained a tumour which was implanted into the anterior chamber of a guinea-pig's eye.

In this experiment, therefore, 5 out of 8 eggs contained small tumours.

*Experiment* 4.—Five 13-day embryonated eggs were inoculated each with 0·02 ml. 24 hours old filtrate prepared by grinding up liver tumour tissue in a glass homogenizer, centrifuging, and filtering, using a Seitz type filter and Ford's " sterimat " grade G.S. The filtrate was inoculated on to the chorioallantois in the usual way, and the eggs sealed with paraffin wax and returned to the incubator.

They were opened on the 5th day after inoculation. Slight opacities were noticed in the membranes, which were not quite so flexuous as normal. Subsequent histological examination, however, showed little abnormality apart from some slight ectodermal proliferation.

*Experiment* 5.—Ten 5- to 6-day-old fertile eggs were implanted on 18.iv.47 with small fragments of liver carcinoma from Case 18. A liver fragment and some of the saline in which it was minced, were placed into broth which on the following day was found to be sterile.

On the 20th of the month 2 embryos were found to be dead. One showed an implanted fragment adhering to the membrane, which was taken for histological examination.

Two further embryos were dead on the 23rd. Both had tumours growing on the membrane. One egg with a living embryo was also opened but there was no sign of a tumour.

The remaining eggs were opened on the 30th. Of the 5, 4 had small growths in the membrane—disappointingly small considering the cultivation period (12 days). Three of these were removed, dissected away from the membranes and reimplanted into two 7-day-old embryonated eggs. The remaining 1 was taken for histological examination. On 10.v.47 the 2 eggs were opened and 1 contained a small dark green growth 2 × 2 × 2 mm. in size. Further subcultivation was not attempted.

*Experiment* 6.—A biopsy was performed on a duck with liver carcinoma (Case 20). The tumour tissue was crushed off the margin of the liver using an emasculator for caponizing cockerels. Haemorrhage was controlled with saline packs and adrenaline. Fragments of tissue were immediately implanted into 5 eggs, 4 containing $10\frac{1}{2}$-day-old embryos, and 1 being 9 days.

The eggs were opened 9 days later; 4 contained tumours. The largest of these (6 mm. diameter) was divided into 4, and reimplanted into 4 eggs of 8 days' incubation. The remainder was taken for histological study. The eggs containing the second passage implants were opened 10 days later, and 3 contained small tumours between 2–3 mm. diameter.

Later a further biopsy on Case 20 provided material to implant into three 8-day embryonated eggs. The duck died near the termination of the operation owing to too deep anaesthesia. The eggs were examined 10 days later, and 2 contained small tumours, about 3 mm. diameter, surrounded as usual by an opaque area.

(3) *Cultivation in the anterior chamber of the eye.*

*Experiment* 7.—Using the technique described by Greene (1941), small tumour fragments from a duck liver (Case 16) were introduced into the anterior chamber of a guinea-pig's eye. Fourteen days later, the eye was examined with the aid of an ophthalmoscope and 2 small brownish-green growths were observed, 1 on the upper pupillary border, and 1 laterally placed. The animal was destroyed on the 18th day of implantation and one of the growths measuring 4 × 3 × 2 mm. removed and implanted into three 9-day embryonated eggs, which upon examination on the 18th day of inoculation showed no tumours on the membrane. The eye was prepared for histological examination of the remaining tumour.

*Experiment* 8.—A small liver carcinoma grown for 7 days on the chorio-allantois was implanted into the anterior chamber of the eye of a guinea-pig. It failed to grow and was eventually absorbed.

*Experiment* 9.—This was a repeat of Experiment 7. A fragment of liver tumour obtained at biopsy (Case 20) was introduced into the anterior chamber as before. The implant failed to grow and was rapidly absorbed.

(4) *Direct transmission experiments* (*homologous*).

*Experiment* 10.—A Khaki Campbell duck, 2 months old, had its liver exposed by intercostal incision, and was given an intra-hepatic injection of 1·0 ml. suspension of liver carcinoma in saline. The original tumour was obtained from Case 13. Sixteen months later the duck was killed and found to have typical liver tumours, which proved to be hepatocellular carcinoma. There were no metastases.

*Experiment* 11.—A 5-month-old Khaki Campbell duck was given an intra-hepatic injection of 1·0 ml. suspension prepared from a liver carcinoma obtained from Case 16. The material used was not more than 1½ hours old. The duck was killed at the end of 16 months, and the liver contained numerous tumours which histologically were cholangiocellular carcinomas. Again there were no metastases.

*Experiment* 12.—Representative portions of liver carcinoma from Case 18 were injected as a suspension in normal saline into the following Aylesbury ducklings (it was not possible to get Khaki Campbell ducklings at short notice):

(1) Four 3-day-old ducklings received 1·0 ml. into the peritoneal cavity.

(2) Four received 0·5 ml. intravenously.

(3) Four received 0·5 ml. intramuscularly. (Actually the injection was practically subcutaneous, the pectoral muscles being poorly developed at this age.). This latter group also received 1·0 ml. cell-free filtrate prepared from the tumour into the peritoneal cavity.

One duckling in group (1) died the following day. Two were destroyed 2 months later and showed no lesions upon post-mortem examination.

In group (2) 2 were destroyed 2 months later and showed no lesions.

In group (3) and in the remainder of groups (1) and (2) the ducks were destroyed 14 months later and upon post-mortem examination showed no abnormality.

*Experiment* 13.—Fragments of liver tumour obtained at biopsy from Case 20 were implanted by means of a wide bore hypodermic needle into the liver of another Khaki Campbell duck, exposed as before by intercostal incision. The bird was then 8 months old. Fourteen weeks later it was observed to be weak, losing weight, and to have an enlarged abdomen. It was destroyed and examination showed a large liver tumour weighing 280 g. with metastases in the lungs, kidney and ovary.

Histological examination of this tumour showed it to be a hepatocellular carcinoma. The original tumour (Table II) was a mixed type, but with cholangiocellular metastases.

TABLE II.—*Metastatic Spread of the Duck Liver Carcinoma.*

| Case No. | Nature of primary tumour. | Organs involved. | Nature of metastasis. |
|---|---|---|---|
| 6 | Liver cell carcinoma | Intestine and ovary | Liver cell carcinoma. |
| 9 | Mixed (liver and bile duct) | Ovary and kidney | ,, ,, |
| 10 | Mixed | Ovary | Bile duct ,, |
| 12 | Liver cell carcinoma | ,, | Liver cell ,, |
| 15 | Bile duct ,, | Lungs and kidney | Bile duct ,, |
| 18 | Mixed | Lungs | ,, ,, |
| 19 | Liver cell carcinoma | Lungs, ovary and kidney | Liver cell ,, |
| 20 | Mixed | Lungs and ovary | Bile duct ,, |
| 21 | Liver cell carcinoma | ,, ,, | Liver cell ,, |
| 22 | ,, ,, | Lungs, ovary, kidney and spleen | ,, ,, |

(5) *Direct transmission experiment* (*heterologous*).

In view of the transmissibility of at least 1 tumour from 1 species of bird to another, i.e. the Fujinami sarcoma of fowls, which has been transmitted to ducks, it was considered worthwhile attempting a similar experiment with the duck liver carcinoma.

*Experiment* 14.—Three 6-week-old Brown Leghorn chickens each received 1·0 ml. tumour suspension into the peritoneal cavity, and 1·0 ml. into the left pectoral muscle. The original tumour was from duck Case 18.

On the 3rd day 1 chicken was found in a dying condition and was killed. The pectoral muscle contained a small greenish-brown growth which was taken for section. This was approximately 66 hours subsequent to injection. No detectable growth was found in the peritoneal cavity.

A 2nd chicken was found dead, 6 days subsequent to injection. There were small fusiform tumours in the pectoral muscle and in the peritoneum.

A 3rd chicken survived and was destroyed 2 months later. No sign of tumour growth was found.

*Histology of cultivated and transplanted tumours.*

The structure of the implants on the chorioallantoic membrane is typically that of the original carcinoma (Fig. 11). There seems to be no tendency to revert to a more undifferentiated type. The liver-cell tumour forms solid cylinders each surrounded by ramifying reticulum fibrils (Fig. 12). The tumour is well supplied with blood vessels derived from the chorioallantois. Mitotic division is frequently seen, but some areas, probably those not vascularized in time, are undergoing necrobiosis, the nuclei showing pyknosis, karyorrhexis and karyolysis. Such areas are infiltrated with phagocytic cells such as macrophages. The capsule is composed of fibroblasts derived from the mesenchymal tissue of the chorioallantoic membrane. It is usually infiltrated by pseudo-eosinophils, and may contain epithelial elements resembling bile ducts.

When a portion of such a tumour is transplanted a second time, the resultant growth is usually small and contains considerable necrotic areas. In those that survive, however, the same structure is apparent, but the bile duct elements, if present, appear to persist longer than the liver cell moiety.

The bile duct type (Fig. 13) is characterized by epithelial cells arranged in the form of ducts or villiform structures, which may take on a cystic appearance.

It has already been shown that the original liver tumour in the duck often varies a great deal in structure in different parts. It is obvious, therefore, that the cultivated tumours will also vary according to the site from which they were taken. This possibly accounts for the differences between the original tumour and the cultivated or transplanted tumours shown in Table III.

TABLE III.—*Nature of Cultivated and Transplanted Duck Liver Carcinoma.*

| Case No., Exp. No. | Diagnosis of original tumour. | Diagnosis of cultivated tumour (C.A. membrane). | Diagnosis of subcultivated tumour (C.A. membrane). | Diagnosis of transplanted tumour (homoplastic and heteroplastic). |
|---|---|---|---|---|
| 16 (1, 5) | L.C. carcinoma | L.C. carcinoma | .. | L.C. carcinoma |
| 18 (3, 11) | Mixed type | B.D. ,, | Necrotic | Mixed type |
| 20 (4, 10) | ,, ,, | (*a*) L.C. carcinoma | L.C. carcinoma | .. |
| | ,, ,, | (*b*) Mainly L.C. but few bile ducts | .. | L.C. carcinoma |

Key : L.C. = Liver cell.
B.D. = Bile duct.
Mixed = Both liver and bile duct elements present.

*Histology of chorioallantoic lesions.*

In every instance where a liver tumour was successfully cultivated on the chorioallantois, a zone of opacity of varying extent developed in the membrane adjacent to the tumour (Fig. 9). This area was only obvious when the membrane and growth were examined against a dark background. Fig. 10 shows the same tumour-bearing membrane photographed against a white background, and the lesions are then barely perceptible.

Upon histological examination, an interesting picture is revealed. The membrane adjacent to the tumour is thickened and very vascular. The thickening is due in the first place to a pronounced proliferation of the ectodermal cells, which become grossly hypertrophied and vacuolated. They measure from 15–30 $\mu$ in diameter (Fig. 14). The nuclei of these cells are irregular and densely staining. Fine cytoplasmic fibrils occupy the cell body, which has a well-defined outer membrane. Eosinophilic masses occur in some cells, resembling to some extent the inclusion bodies in a fowl-pox lesion of the chorioallantois.

Secondly, there is a proliferation of connective tissue in the membrane. Lying enclosed in this stroma are epithelial structures resembling the " cell nests " of squamous-cell carcinoma. These are eosinophilic concentric bodies whose outer coats are composed of flattened epithelial cells derived from a down-growth of the chorioallantoic ectoderm. Passing towards the centre of the structure, these cells become hyalinized and lose their nuclei by fragmentation. The centres of the bodies are composed of a mass of dead, occasionally vacuolated cells (Fig. 15, 16).

Table IV shows that tumour implants stimulated ectodermal proliferation to

TABLE IV.

| Material. | Virus. | Experiment No. | Reaction of adjacent membrane. Ectodermal proliferation. | " Cell nest " formation. |
|---|---|---|---|---|
| Duck liver carcinoma | ? | 3 | + | + |
| Filtrate | ,, | 4 | + | – |
| Duck liver carcinoma | ,, | 5 | + | + |
| ,, ,, | ,, | 5 (subcultivation) | + | + |
| ,, ,, | ,, | 6 | + | + |
| ,, ,, | ,, | 6 (subcultivation) | + | + |
| ,, ,, | ,, | 6 (2nd biopsy) | + | + |

the extent of " cell nest " formation. Filtrate on the other hand, simply caused ectodermal proliferation.

*Histology of transplanted tumours (heteroplastic grafts).*

In Experiment 14, duck liver carcinoma suspension was injected by various routes into young Brown Leghorn chickens, where the tumour grew successfully up to 6 days.

The nodule removed from the pectoral muscle after 66 hours shows groups of viable carcinoma cells, forming distinct glandular acini. Some cells are actively dividing, and the graft appears to be infiltrating the muscle (Fig. 17). The tumour tissue is vascularized and in places a connective tissue stroma contains epithelial structures resembling bile ducts. There is some necrosis of the muscle adjacent to the graft, probably due to trauma following injection, and an early host reaction around the foreign cells is indicated by the presence of infiltrating histiocytes, macrophages, pseudo-eosinophils and a few lymphocytes.

In the peritoneum, the host reaction appears to be much more vigorous. The nodule shows a dense connective tissue base (thickened peritoneum), growing from which is a mass of vascularized lymphoid tissue containing a few islands of surviving liver cells (Fig. 18).

The 6-day graft (Fig. 19) shows a bile duct type of tumour, with a villiform structure. It is still actively growing and infiltrating the muscle, but there are only a few surviving liver cells in the form of acini, and these are engulfed in a massive lymphocytic infiltration.

In Experiment 7, fragments of liver carcinoma (Case 16, liver cell type) were implanted into the anterior chamber of the eye of a guinea-pig. Two tumours grew. On the 18th day the animal was destroyed and the eye opened. One tumour measuring 4 × 3 × 2 mm. was used for further transplantation experiments and the other smaller growth was left *in situ*, and the eye prepared for histological examination.

This shows a small tumour composed mainly of an undifferentiated mass of epithelial cells attached to the iris (Fig. 20). Some attempt to form cord-like structures may be seen. The growth is surrounded by a zone of fibroblasts and is in parts infiltrated with lymphoid cells. Vascular connections with the capillaries of the iris can be seen.

### DISCUSSION.

In a previous communication, Campbell (1949), reported the results obtained by implanting various spontaneous and chemically induced chicken and mammalian tumours into the fertile hen egg. Two main sites were used, namely the yolk-sac and the chorioallantoic membrane. Yolk-sac cultivation did not give satisfactory results, this also being the experience of Twombly and Meisel (1946); although Taylor, Thacker and Pennington (1942), and several other workers claimed a high perentage of intravitelline growths with the particular tumours used. These were mainly mouse and rat mammary carcinomata, and the poor results obtained by others, using different tumours, seems to indicate that the environment provided by the chick embryo yolk-sac is peculiarly suitable for the metabolic requirements of mammary cancer cells.

On the other hand, most of the tumours implanted on the chorioallantois grew successfully and were readily visible, and the death rate of embryos was considerably lower than after yolk-sac injection. The sudden occurrence of a number of cases of hitherto unrecorded primary carcinoma of the duck's liver provided material for cultivation by various methods, with the main object of searching for a transmissible factor or virus. The chorioallantoic method of cultivation, therefore, seemed particularly suitable, and was extensively used.

There appear to be two possible explanations of the etiology of this condition. Firstly, the disease might be associated with a transmissible factor. It is known that the mouse mammary cancer factor of Bittner is transmitted from mother to offspring by the milk, and this is one of the very few known instances of a tumour of epithelial nature associated with a virus-like principle. It was felt that the duck liver carcinoma might also contain a factor transmissible probably *via* the egg, which might be capable of propagation in the same manner as the milk factor of mice.

Experiments designed to test this theory, however, have given negative results as far as transmission by cell-free material is concerned. The only evidence for an associated virus has been that obtained from the cultivation of the tumour on the chorioallantoic membrane, where in every instance typical lesions developed in the ectoderm adjacent to the implant, or subsequent to inoculation with filtrates of the tumour. Also, yolk-sac injections resulted in

---

EXPLANATION OF PLATES.

FIG. 1.—Liver, and cut surface of one lobe, showing a hepatocellular carcinoma of a Khaki Campbell duck. 4/5th natural size.
FIG. 2.—Metastases of hepatocellular carcinoma in the lungs, ovary and kidneys of a Khaki Campbell duck. 4/5th natural size.
FIG. 3.—Hepatocellular carcinoma. Khaki Campbell duck. H. & E. × 145.
FIG. 4.—Detail of duck hepatocellular carcinoma. H. & E. × 660.
FIG. 5.—Anaplastic type of duck hepatocellular carcinoma. H. & E. × 690.
FIG. 6.—Cholangiocellular carcinoma. White Campbell duck. H. & E. × 145.
FIG. 7.—Metastasis of bile duct moiety of a primary mixed tumour to the lung of a Khaki Campbell duck. H. & E. × 56.
FIG. 8.—Embolus of liver carcinoma cells within vein of intestinal wall, Khaki Campbell duck. H. & E. × 660.
FIG. 9.—Duck liver carcinoma cultivated on the chorioallantoic membrane. Note surrounding zone of reaction. × 2.
FIG. 10.—The same, photographed against a white background. The reaction zone is not now apparent.
FIG. 11.—Structure of duck hepatocellular carcinoma growing on the chorioallantois. H & E. × 56.
FIG. 12.—Detail of duck hepatocellular carcinoma growing on the chorioallantois showing reticulum. Wilder's method. × 260.
FIG. 13.—Cholangiocellular carcinoma of the duck, growing on the chorioallantois. The tendency to form duct-like structures is well shown. H. & E. × 56.
FIG 14.—" Ballooned " ectodermal cells of the chorioallantois, adjacent to an implanted duck liver carcinoma. H. & E. × 530.
FIG. 15.—" Cell-nests " in the chorioallantois, adjacent to implanted liver carcinoma. Mallory's stain. × 56.
FIG. 16.—Detail of a typical " cell-nest " in the chorioallantois. Mallory. × 260.
FIG. 17.—Growing periphery of duck liver-cell carcinoma in pectoral muscle of a young chicken 66 hours after implanting. H. & E. × 260.
FIG. 18 —Lymphocytic reaction to the duck liver carcinoma, 6 days after implantation into the peritoneum of a chicken. H. & E. × 260.
FIG. 19.—Cholangiocellular carcinoma of the duck, infiltrating pectoral muscle of a chicken, 6 days subsequent to implantation. H. & E. × 56.
FIG. 20.—The duck liver-cell carcinoma growing in the anterior chamber of a guinea-pig's eye. A tendency to grow in cords is to be noted. H. & E. × 260.

1

2

Campbell.

3

4

5

6

7

8

Campbell.

15

16

17

18

19

20

Campbell.

the development of haemorrhages and death of the embryo, and the allantoic fluid from such embryos caused the death of embryos upon a second passage into eggs.

With regard to the ectodermal lesions, it has been shown that they reslut from the implantation of most tumours, also embryonic tissue, but not from normal adult tissues. It was suggested that these findings might indicate the presence of growth-stimulating substances in neoplastic and embryonic tissues, and supporting evidence for this hypothesis was produced (Campbell, 1949). It is clear, therefore, that the ectodermal lesions described cannot be regarded as evidence for the presence of a virus *per se*, although the lethal effect on embryos of injecting tumour material into the yolk-sac could conceivably be interpreted as such.

It is possible that if a virus is present in these tumours, it is not capable of demonstration by the methods used. It is well known that Rous I virus undergoes phases of inactivity when for a period it is difficult to induce tumours by filtrates. Similar inhibitors might be responsible for the failure to transmit the duck tumour by filtrate. The only successful transmissions were accomplished by the direct implantation of tumour tissue into the livers of recipients of the same breed. It is not surprising that the tumour could not be transmitted to young chickens, but the survival of these heterologous grafts for at least 6 days in the muscle and peritoneum of chickens is rather remarkable.

The second possible explanation of this apparent outbreak of liver cancer, is a genetic one. The condition was only encountered in a single breed of ducks, with the one exception where the bird was of a closely related strain. As far as could be ascertained the individuals in the small flocks involved bore either the sister or cousin relationship to each other, whilst the tracing of 2 separate affected flocks to a common source in the North of England may be a highly significant fact.

Turning to the spread of the tumour in the body, Cases 6 and 22 are of some interest owing to the sites of secondary deposits, respectively intestine and spleen. In the literature on primary liver cell carcinoma in the human subject, splenic metastases are mentioned but rarely. Cappell (1925) described a case of multiple microscopic metastases in the spleen in a case of liver cell carcinoma and remarks on the smallness of these secondaries in comparison with the large growths in the lungs, bronchi, pleura, heart and brain ; but he offers no explanation as to how the spleen becomes involved. There appear to be 2 possibilities, firstly, a retrograde spread along the hepatic vein to the coeliac vein, and thence back to the spleen, or, secondly, a complete circuit of the vascular system through the pulmonary circulation and so to the spleen *via* the splenic artery. In the one case encountered in the duck, pulmonary metastases occurred, and so this second hypothesis may be the correct one. Once the lungs are involved the tumours there may also metastasize ; tumour cells or emboli could then reach the spleen without much difficulty. A direct spread *via* the lungs seems unlikely in view of the formidable barrier offered by the pulmonary capillaries.

Fox and Bartels (1928) favour the view that in the human subject the spleen and mesenteric veins may be invaded by retrograde extension along the portal vein. In Case 6, besides serosal implantation on the wall of the intestine, tumour emboli were found within the veins of the gut wall, and their presence could be accounted for in this manner.

### SUMMARY AND CONCLUSIONS.

A hitherto unrecorded neoplastic disease affecting the liver of the 2–3 year old female Khaki Campbell duck has been investigated, and details are given of the history, symptoms, post-mortem findings and pathology, together with experimental data resulting from cultivation by various *in vivo* methods.

It is concluded that while an associated transmissible factor cannot be definitely excluded, certain evidence has been collected that a genetic factor may probably be involved in its etiology.

I wish to express my gratitude to Professor A. Murray Drennan of the Pathology Department, University of Edinburgh, for his interest in this work, and for numerous suggestions as to the manner of development of the investigation.

All the fertile eggs were obtained from Dr. A. W. Greenwood's flock of Brown Leghorns, and I have pleasure in recording my appreciation of his co-operation in this respect, and for much help in other directions.

The histological preparations are the work of Messrs. Cockburn and MacKenzie.

Part of the expenses incurred by this work was borne by the British Empire Cancer Campaign.

The colour photographs are the work of Mr. Richard Hood, Head Technician at the Royal (Dick) Veterinary College, Edinburgh.

### REFERENCES.

Beveridge, W. I. B., and Burnet, F. M.—(1946) *Spec. Rep. Ser. med. res. Coun. Lond.*, No. 256.

Campbell, J. G.—(1946) *Nature*, **158**, 711.—(1949) *Brit. J. Cancer*, **3**, 72.

Cappell, D. F.—(1925) *J. Path. Bact.*, **28**, 129.

Fox, R. A., and Bartels, G. W.—(1928) *Arch. Path.*, **6**, 228.

Greene, H. S. N.—(1941) *J. exp. Med.*, **73**, 461.

Taylor, A., Thacker, J., and Pennington, D.—(1942) *Science*, **96**, 342.

Twombly, G. H., and Meisel, D.—(1946) *Cancer Res.*, **6**, 82.